# Workplace loneliness mediates the relationship between perceived organizational support and job performance: Differing by extraversion

**DOI:** 10.3389/fpsyg.2023.1058513

**Published:** 2023-03-13

**Authors:** Guomei Tian, Tingting Liu, Ruo Yang

**Affiliations:** ^1^School of Business, Xi’an University of Finance and Economics, Xi'an, Shanxi, China; ^2^School of Management, Shanghai University of International Business and Economics, Shanghai, China; ^3^School of Management, Chengdu University of Information Technology, Chengdu, Sichuan, China

**Keywords:** perceived organizational support, workplace loneliness, extraversion, job performance, conservation of resources theory, social cognitive theory

## Abstract

This study investigated the mediating role of workplace loneliness relating perceived organizational support to job performance, as well as the moderating role of extraversion in such relationship. 332 full-time Chinese employees from various enterprises voluntarily participated in the two-wave surveys *via* either paper-and-pencil or online survey conducted at Credamo and Tencent Questionnaire website. Hierarchical regression and bootstrapping analyses were employed to examine the hypotheses. Results indicated that workplace loneliness partially mediates the linkage between perceived organizational support and job performance; extraversion serves as a moderator in the relationship between workplace loneliness and job performance, as well as the mediating role of workplace loneliness linking perceived organizational support to job performance, such that the relationship is stronger when extraversion is high. Supplementary analyses revealed that social companionship, but not emotional deprivation, serves as a mediator in the relationship between perceived organizational support and job performance; extraversion enhanced the direct influence of social companionship on job performance, as well as the indirect influence of perceived organizational support on job performance *via* social companionship. Theoretical and practical implications are discussed.

## Introduction

Workplace loneliness, defined as one’s perceptions of relational deficiencies in the workplace (Wright et al., 2006), has been found to be increasingly prevalent in recent years (Ozcelik and Barsade, 2018; Anand and Mishra, 2021). According to *Loneliness Economy White Paper 2017*, more than 60% of respondents indicated some degree of workplace loneliness (Wang and Liu, 2021). Much work has been conducted concerning its detrimental effects on individuals and enterprises. Specifically, workplace loneliness negatively relates to employees’ well-being (Mohapatra et al., 2020), creativity (Peng et al., 2017), job performance (Lam and Lau, 2012; Ozcelik and Barsade, 2018), and organizational citizenship behaviors (Lam and Lau, 2012; Firoz and Chaudhary, 2021), and positively associates with work–family conflict (Firoz and Chaudhary, 2021).

Given the negative effects of workplace loneliness, much attention has been paid to its antecedents. Prior studies indicated that job characteristics, such as job autonomy (Wang and Liu, 2021) and job insecurity (Andel et al., 2021), leadership factors, including paternalistic leadership (Öge et al., 2018) and transformational leadership (Kloutsiniotis et al., 2022), and social interaction variables, such as leader-member exchange, trust, and communication frequency (Arslan et al., 2020) are closely correlated to workplace loneliness. This study focuses on the effect of perceived organizational support (one remarkable organizational factor) on workplace loneliness, and more importantly, the mediating role of workplace loneliness linking perceived organizational support to job performance.

According to the conservation of resources (COR) theory, people strive to obtain and maintain resources, including both personal resources and organizational resources that are beneficial for them to achieve goals (Hobfoll, 1989; Hobfoll and Shirom, 2001; Hobfoll et al., 2003). Hobfoll (2011) further claimed that people’s resources do not exist independently but travel in caravans for both individuals and enterprises. As shown in prior research, in the organizational context, individuals who have been well supported are more likely to construct or receive high leader-member exchange (Wayne et al., 1997, 2002). In the present article, we aim to examine the influence of perceived organizational support on workplace loneliness.

The COR theory also posits that potential resource losses or gains would influence one’s emotions, thus leading to poorer or better performance. Drawing on this theory, much work has been conducted regarding the mediating role of emotions linking personal or organizational resources to job performance. For example, Guan et al. (2014) found that job satisfaction serves as a mediator linking perceived organizational support to job performance. Moreover, workplace loneliness serves as a mediator in the relationship between transformational leadership and burnout (Kloutsiniotis et al., 2022). Based on this premise, we propose that workplace loneliness would function as a mediator in the bonds between perceived organizational support and job performance.

Moreover, according to the social cognitive theory (SCT), human activity is jointly influenced by employees’ behavior, their cognition and personalities, and external circumstances (Bandura, 1991; Bandura, 2001). In this regard, even though individuals have the same emotional experience, their perceptions or reactions may differ. SCT was widely employed to explore the moderating effect of personal traits in one’s response to emotions. For example, Wanberg et al. (2020) discovered that extraversion serves as a moderator in the relationship between participating in the networking intervention and postintervention in a field experimental study. Similarly, core self-evaluations and conscientiousness are indicated to moderate the indirect influence of supervisor bottom-line mentality on social undermining *via* employee bottom-line mentality (Greenbaum et al., 2012). In the present research, we examine the moderating role of extraversion in the relationship between workplace loneliness and job performance. As extraverts tend to attach more importance to close interpersonal relationships and have higher intention to display warmth, affection, and friendliness (Hogan, 1982; Depue and Collins, 1999), they may be more frustrated when suffering from poor social ties in the workplace.

**Figure 1:**
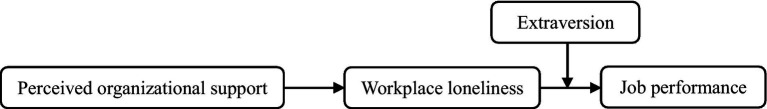
The moderated mediation model in this study.

In summary, integrating the COR and SCT theories, we proposed a moderated-mediation model explaining how and for whom perceived organizational support may promote job performance, as shown in Figure 1. The contributions of the present research are threefold. Firstly, we contribute to the limited literature on the antecedents of workplace loneliness. Given that existing research on the antecedents of workplace loneliness mainly focuses on job characters and interactions with leaders (e. g., Öge et al., 2018; Andel et al., 2021). As we know, few studies have examined the influence of organizational factors such as perceived organizational support, which is the focus of the present research. Our study contributes to a new view on the antecedents of workplace loneliness.

In addition, we contribute to the nascent knowledge on the association between perceived organizational support and job performance by exploring the mediating effect of workplace loneliness. Although previous studies have identified some underlying mechanisms on how perceived organizational support influences job performance (e. g., Rubaca and Majid Khan, 2020; Zhang et al., 2021), they mainly focus on the mediating role of positive emotions or psychological states. Whether negative emotions (one of which is workplace loneliness) would serve as a mediator in such relationship has not been examined yet.

Finally, we tested the moderating role of extraversion in the workplace loneliness-job performance association, which extends our understanding of the boundary conditions for the effect of workplace loneliness. Although prior research has indicated that consciousness and need to belong moderate the influence of workplace loneliness on its outcomes (Hu et al., 2021; Basit and Nauman, 2023), it remains to be examined whether extraversion would function as a moderator in such relationship. We address this issue by examining the moderating role of extraversion in the relationship between workplace loneliness and job performance, and the indirect influence of perceived organizational support on job performance *via* workplace loneliness. This study extends SCT to the workplace loneliness literature, indicating that perceived organizational support inhibits workplace loneliness, thus leading to better job performance, and the strength of such relationships depends on the level of one’s extraversion.

## Literature review and hypotheses

### Perceived organizational support, workplace loneliness, and job performance

COR theory posits that both personal resources and organizational resources are beneficial for employees to achieve their goals in the work context (Hobfoll, 1989; Hobfoll and Shirom, 2001). As one form of organizational resources, perceived organizational support, referring to an employee’s overall perceptions of the extent to which their contributions are valued and their interests or well-being are acknowledged by the organization they work in, provides aids to workers in terms of equipment, funding, ideas, and physical assistance in the workplace (Eisenberger et al., 1986, 1997; Rhoades and Eisenberger, 2002). Individuals with high perceived organizational support tend to believe that their need for work or life will be satisfied and they are well respected or recognized by the organization (Eisenberger et al., 1986, 1997), and, in turn, display increased work engagement (Karatepe and Aga, 2016; Tan et al., 2020; Xu et al., 2022), affective commitment (Guan et al., 2014; Nazir et al., 2019), and better job performance (Chiang and Hsieh, 2012; Guan et al., 2014). There is extensive evidence indicating the positive association between perceived organizational support and job performance employing different sources of job performance data. For example, Gillet et al. (2013) found that perceived organizational support positively relates to one’s self-reported job performance; this relationship was also certificated in a survey on employees in Taiwan hotels (Chiang and Hsieh, 2012). In a study on frontline bank employees, Karatepe and Aga (2016) found that perceived organizational support positively correlates with supervisor-rated job performance. The relationship was also confirmed in a study employing job performance data from HR archival records (Tian et al., 2021).

Moreover, as one kind of external coping resource, perceived organizational support responds to the socio-emotional needs of employees (Kraimer et al., 2001), it is supposed to influence one’s emotions or psychological condition. Much work has been conducted on the positive emotions or psychological conditions provoked by perceived organizational support including resilience (Zhang et al., 2021), psychological safety (Xu et al., 2022), meaningfulness (Guan and Frenkel, 2021), and job satisfaction (Guan et al., 2014; Rubaca and Majid Khan, 2020). In addition, drawing on the COR theory, people’s resources including personal and organizational resources co-exist in ecological conditions that either foster or limit resource creation and sustenance (Hobfoll, 2011). As employees with high perceptions of organizational support tend to perform well, it is more likely that they will be better expected by their leaders, thus contributing to better leader-member exchange (Wayne et al., 1997, 2002). For example, Wayne et al. (1997) found that perceived organizational support is positively related to leader-member exchange in a study on two metal fabricating plants. Similarly, the relationship was confirmed in the work of Wayne et al. (2002). Considering that workplace loneliness concerns employees’ perceptions of insufficient interpersonal interactions with their colleagues those they work with or enterprises they work for either quantitatively or qualitatively (Wright et al., 2006), perceived organizational support contributes to high-quality leader-member exchange. It is reasonable to predict that perceived organizational support would be negatively related to workplace loneliness.

From a COR perspective, employees suffering from workplace loneliness usually get into a stressful situation, and they tend to decrease their affective commitment (Ozcelik and Barsade, 2018) or work engagement (Basit and Nauman, 2023) so as to preserve their personal resources, which ultimately result in more social cyberloafing behaviors (Hu et al., 2021) and poor job performance (Ozcelik and Barsade, 2018; Abelsen et al., 2021). Prior research has extensively confirmed the negative relationship between workplace loneliness and job performance. For example, Ozcelik and Barsade (2018) found that employees’ self-reported workplace loneliness negatively links to supervisor-rated job performance. Workplace loneliness also negatively relates to task performance and contextual performance (Abelsen et al., 2021). More importantly, the work of Wax et al. (2022) indicated that workplace loneliness has a detrimental influence on self-reported job performance.

Besides the direct relationship of perceived organizational support-workplace loneliness and workplace loneliness-job performance, we also examine the mediating role of workplace loneliness. According to the COR theory, job resource can accumulate and therefore contribute to positive outcomes (Hobfoll, 1989; Hobfoll and Shirom, 2001). In this vein, as one kind of organizational resources in the workplace, perceived organizational support is supposed to contribute to accumulation of personal resources, which, in turn, lead to positive outcomes. For example, the mediating role of job satisfaction in associating perceived organizational support with contextual performance has been confirmed (Rubaca and Majid Khan, 2020). Similarly, Guan and Frenkel (2021) found that meaningfulness functions as a mediator in the association between perceived organizational support for strength use and thriving at work. Considering that perceived organizational support contributes to one’s satisfaction of their need for affiliation and workplace loneliness is negatively related to job performance, we argue that workplace loneliness would serve as a mediator in the relationship between perceived organizational support and job performance.

Consequently, perceived organizational support would be negatively related to workplace loneliness, and workplace loneliness would serve as a mediator in the association between perceived organizational support and job performance. Therefore, we predict that:

*H1*: Perceived organizational support is negatively linked to workplace loneliness.

*H2*: Workplace loneliness functions as a mediator in the relationship between perceived organizational support and job performance.

### The moderating role of extraversion

We further propose that the negative effect workplace loneliness exerts on job performance varies for people according to whether they have high or low levels of extraversion. SCT posits that human behavior is extensively motivated and regulated by both self-generated and external sources of influence (Bandura, 1991). One of the major self-regulative mechanisms operates in the judgment of one’s behavior according to personal standards. Owing to the variances in personal standards among people of different personalities, their responses to the same events or emotional experiences may vary. As indicated in the work of Beck et al. (1983), the influence of interpersonal rejection or loss on depression varies for employees who are high or low in sociotropy or social dependence. Similarly, the work of Mohapatra et al. (2020) revealed that self-esteem serves as a moderator in the bonds between workplace loneliness and work-alienation. In the present, we aim to focus on the moderating role of extraversion.

Extraversion is a dispositional tendency to seek out and enjoy frequent and intimate interpersonal interactions, and to be self-confident, active, and dominant (Barrick and Mount, 1991). The moderating role of extraversion in employees’ response to their perceptions or experiences at work has been confirmed in previous research. For instance, extraversion serves as a moderator in the relationship between span of control and the quality of leader-member exchange, such that the relationship is stronger when extraversion is high (Schyns et al., 2012). Similarly, prior research indicated the moderating role of extraversion in the relationship between agentic threats and support-seeking behaviors (Pow et al., 2017). Moreover, Lajoie et al. (2022) found that a leader’s extraversion functions as an enhancer in the linkage between transformational leadership and follower vitality.

Considering that extraverts have higher need for frequent and intense social interactions (Hogan, 1982; Depue and Collins, 1999), communications with others may help to reduce their anxiety. As shown in an experimental study, extraverts experienced a lower level of anxiety than introverts after communication with a confederate *via* either computer-mediated or face-to-face communication (Rice and Markey, 2009). It is reasonable to predict that, when experiencing poor or deficient social contacts, extraverts may be more anxious or frustrated. In other words, people high in extraversion would be more oppressed by deficient interpersonal relationships. There is some evidence supporting the reverse buffering role of extraversion. In a study on students, Mishra et al. (2018) found that extraverts experienced higher levels of depression than introverts when suffering from loneliness. Thus, we propose that:

*H3*: Extraversion will moderate the negative relationship between workplace loneliness and job performance, such that the relationship is stronger when extraversion is high.

### The moderated-mediation model

When H2 and H3 are integrated, a moderated-mediation pattern is implied: due to the moderating role of extraversion on the association between workplace loneliness and job performance, extraversion is likely to accentuate the indirect influence of perceived organizational support on job performance *via* the mediating role of workplace loneliness. Accordingly, we predict:

*H4*: Extraversion would moderate the mediating effect of workplace loneliness in the association between perceived organizational support and job performance, such that the relationship is stronger for extraverts than introverts.

## Methods

### Participants and procedures

All employees voluntarily participated in the two-wave surveys *via* either a paper-and-pencil or online survey conducted at Credamo or Tencent Questionnaire website. In the first wave, 495 full-time employees from different enterprises reported their demographic information and degree of extraversion. One month later, when the scales of perceived organizational support, workplace loneliness, and job performance were distributed to them, 421 questionnaires were returned. After deleting invalid or impaired responses, the final sample consisted of 332 participants, representing a response rate of 67.07%. It included 206 females (62.05%) and 126 males (37.95%). Fifteen yuan (about $2.35) was given to participants as a compensation for their involvement. The average age and organizational tenure were 30.76 (SD = 5.30) and 5.02 (SD = 3.84), respectively; 82.23% had a bachelor or graduate degree and 56.63% were ordinary employees. More than one-half of participants (54.22%) worked in private-owned enterprises, followed by employees worked in state-owned enterprises (21.08%) and public institutions (10.84%). Approximately one-quarter of participants (25.90%) worked in high-tech and other industries (28.31%). Participants working in the traditional industries, financial industry, and service industry were 21.08, 8.73, and 7.83%, respectively.

### Measures

Participants were asked to report the extent to which these items are accurate in describing their perceptions at work, using a 5-point Likert scale, ranging from “1 = it does not describe me at all” to “5 = it accurately describes me.” We conducted translation and back-translation procedures (Brislin, 1980) to translate English items into Chinese.

#### Perceived organizational support

We employed the 8-item scale developed by Eisenberger et al. (1997) to measure perceived organizational support. A sample was “My organization cares about my opinions.” Item 6 was reverse scored. The reliability value of this scale was 0.84.

#### Workplace loneliness

The 16-item scale developed by Wright et al. (2006) was employed to measure workplace loneliness. It includes two dimensions: emotional deprivation, which refers to the perception of the amount or quantity of one’s relationships at work, and social companionship, defined as the perception of the quality of one’s interpersonal relationships in the workplace. Emotional deprivation includes 9 items (e. g., “I often feel alienated from my co-workers”) and social companionship contains 7 items (e. g., “I often feel disconnected from others at work”). Eight items were reverse scored, two items in the dimension of emotional deprivation, and 6 items from another dimension. The Cronbach’s α was 0.91.

#### Job performance

We employed the scale developed by Janssen (2001) to measure job performance, which contains 5 items. The original version of the scale was used for managers or supervisors to evaluate the performance of his or her subordinates, we adapted this scale for employees to evaluate their job performance. A sample was “I always complete the duties specified in my job description.” Item 3, “I often fail to perform essential duties,” was deleted since its factor loading was less than 0.50. The reliability value of this scale was 0.76.

#### Extraversion

The 8-item scale developed by Benet-Martínez and John (1998) was employed to measure extraversion. Samples are “I like to talk” and “I am outgoing and sociable.” Three items were reverse scored. The Cronbach’s α was 0.81.

#### Control variables

Demographic variables including gender and age were controlled, as they have been indicated to be linked to loneliness (Schmidt and Sermat, 1983). In addition, based on prior research (Ozcelik and Barsade, 2018; Zhang et al., 2021), we also controlled organizational tenure, educational level, and position in the organization. Gender was dummy coded: 0 stands for male and 1 stands for female. Age and organizational tenure were self-reported in years. Educational level was measured in the range of 1–5, representing middle school or below, high school, college, university, and postgraduate, respectively; position in the organization was measured in four categories from 1 to 4, indicating employees, junior manager, middle manager, and senior manager, separately.

## Results

The Harman’s one-factor test was employed to examine the common method bias. The single factor explained 28.21% of the variance, which is lower than 40%, implying that the common method bias is not significant in this study. Both dimensions of workplace loneliness and extraversion were modeled using parcel scores. Each includes two of the original items, except for one item of emotional deprivation and one item of social companionship, which were treated as an independent score so as to restrict the number of indicators owing to limited responses (Little et al., 2002; Matsunaga, 2008).

### Confirmatory factor analysis (CFA)

To evaluate the discriminant validity among variables included in the study, a series of CFAs was conducted before examining the hypotheses, as shown in Table 1. Our hypothesized five-factor model, including perceived organizational support, two dimensions of workplace loneliness (emotional deprivation and social companionship), extraversion, and job performance, was found to adequately fit the data: (*χ*^2^/df = 2.39, RMSEA = 0.065, IFI = 0.916, TLI = 0.903, CFI = 0.915). Moreover, the five-factor model was also found to fit the data significantly better than other alternative models.

**Table 1:** Comparison of alternative models.

Models	Factors	χ^2^/df	Δχ^2^	RMSEA	IFI	TLI	CFI
1	Five factors: POS, ED, SC, EX, JP	2.39		0.065	0.916	0.903	0.915
2	Four factors: POS, ED+ SC, EX, JP	2.86	134.66^**^	0.075	0.885	0.870	0.884
3	Three factors: POS, ED+ SC, EX+ JP	3.96	440.18^**^	0.095	0.815	0.793	0.814
4	Two factors: POS + ED+ SC, EX+JP	5.48	861.85^**^	0.116	0.718	0.687	0.717
5	One factor: POS + ED+ SC + EX+JP	6.37	1109.47^**^	0.127	0.661	0.626	0.659

^**^p < 0.01. POS, perceived organizational support; ED, emotional deprivation; SC, social companionship; EX, extraversion; JP, job performance.

### Hypotheses testing

The means, standard deviations, and correlations among all variables are shown in Table 2.

**Table 2:** Means, standard deviations, and correlations among all variables.

Variables	Mean	SD	1	2	3	4	5	6	7	8
1. Gender	0.62	0.49								
2. Age	30.76	5.30	−0.075							
3. Tenure	5.02	3.84	−0.120^*^	0.636^**^						
4. Education	4.05	0.75	−0.112^*^	0.024	0.048					
5. Position	1.65	0.86	−0.178^**^	0.308^**^	0.374^**^	0.148^**^				
6. POS	3.76	0.62	−0.030	0.026	0.145^**^	−0.072	0.199^**^			
7. WL	2.01	0.51	0.050	−0.053	−0.162^**^	−0.055	−0.119^*^	−0.551^**^		
8. EX	3.44	0.57	−0.175^**^	0.052	0.170^**^	0.035	0.234^**^	0.376^**^	−0.345^**^	
9. JP	4.23	0.54	−0.035	0.090	0.256^**^	0.048	0.135^*^	0.446^**^	−0.459^**^	0.299^**^

*N* = 332, ^**^p < 0.01, ^*^p < 0.05. POS, perceived organizational support; WL, workplace loneliness; EX, extraversion; JP, job performance.

Hypothesis 1 predicted that perceived organizational support would be negatively related to workplace loneliness. Results in Table 2 confirmed the negative linkage between perceived organizational support and workplace loneliness (*r* = −0.551, *p* < 0.01); thus, hypothesis 1 was supported. The hierarchical regressions and bootstrapping analyses were then conducted to test other hypotheses (Baron and Kenny, 1986). The result of the hierarchical regressions is shown in Table 3.

**Table 3:** Results of hierarchy regressions.

Variables	Job performance					
	Model 1	Model 2	Model 3	Model 4	Model 5	Model 6
Gender	0.01	0.00	0.01	0.01	0.03	0.02
Age	−0.15^*^	−0.08	−0.07	−0.10	−0.08	−0.08
Tenure	0.41^**^	0.31^**^	0.27^**^	0.30^**^	0.28^**^	0.28^**^
Education	0.01	0.05	0.03	0.00	0.00	0.00
Position	0.04	−0.03	−0.02	0.02	0.00	0.01
Perceived organizational support		0.39^**^	0.25^**^			
Workplace loneliness			−0.26^**^	−0.39^**^	−0.36^**^	−0.39^**^
Extraversion					0.12^*^	0.16^**^
Workplace loneliness *Extraversion						−0.18^**^
*R* ^2^	0.13	0.27	0.32	0.27	0.28	0.31
Δ*R*^2^	0.13	0.14	0.05	0.14	0.01	0.03
Δ*F*	9.64^**^	62.16^**^	21.76^**^	64.37^**^	5.27^*^	13.88^**^

*N* = 332; ^**^p < 0.01, ^*^p < 0.05.

Hypothesis 2 predicted workplace loneliness would mediate the relationship between perceived organizational support and job performance. As indicated in Table 3, perceived organizational support was positively associated with job performance (*r* = 0.39, *p* < 0.01), and the linkage between them was still significant (*r* = 0.25, *p* < 0.01) when workplace loneliness entered the model, indicating that workplace loneliness partly mediated the positive bonds between perceived organizational support and job performance. Bootstrapping analyses in PROCESS (Hayes, 2013), generating 5,000 samples, were then conducted to further test the hypothesis concerning the mediating role of workplace loneliness. Once again, Hypothesis 2 was confirmed, as the indirect effect of perceived organizational support on job performance *via* workplace loneliness was 0.123 (SD = 0.046), *p* < 0.01, and BC 95% CI = [0.029, 0.214] did not include zero.

**Figure 2:**
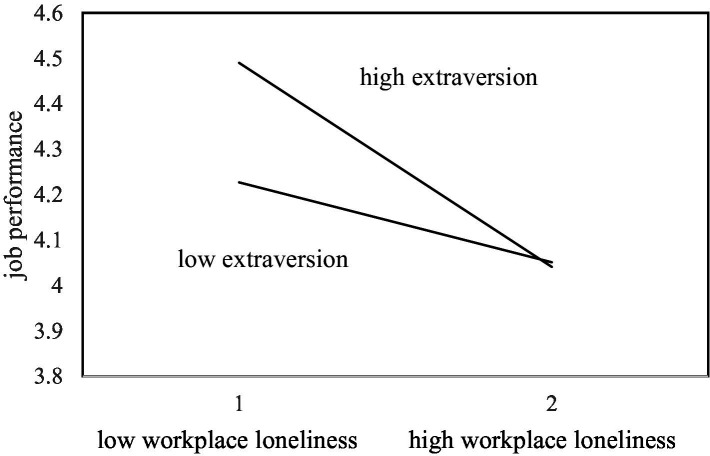
The interactive influence of workplace loneliness and extraversion on job performance.

In hypothesis 3, we predicted extraversion would moderate the linkage between workplace loneliness and job performance. As shown in Table 3, there was a significantly negative influence of the interaction between workplace loneliness and extraversion on job performance (*r* = −0.18, *p* < 0.01). Following Aiken et al. (1991), we explicated the interaction, as shown in Figure 2. This revealed that for participants high in extraversion, workplace loneliness was negatively related to job performance (*r* = −0.443, *p* < 0.01), while for participants low on extraversion, workplace loneliness was also negatively related to job performance (*r* = −0.173, *p* < 0.01), confirming the moderating role of extraversion. Hypothesis 3 was thus supported.

The bootstrapping analyses using Model 14 in PROCESS (Hayes, 2013), generating 5,000 samples, were conducted to examine hypothesis 4 concerning the moderated-mediation model. The results in Table 4 indicate a significant moderated mediation effect (the contrast = 0.118, SE = 0.043, BC 95% CI = [0.028, 0.201], did not include zero). In particular, for individuals low on extraversion, the mediation effect of workplace loneliness in linking perceived organizational support and job performance was not significant (indirect effect = 0.076, SE = 0.043, BC 95% CI = [−0.009, 0.160], included zero); while, for individuals high in extraversion, the indirect effect of perceived organizational support on job performance *via* workplace loneliness was significant (indirect effect = 0.195, SE = 0.058, BC 95% CI = [0.082, 0.307], did not include zero). Thus, Hypothesis 4 was supported, as the mediating effect of workplace loneliness linking perceived organizational support to job performance varies for participants high or low on extraversion.

**Table 4:** Results of moderated mediating role of extraversion in the indirect influence of perceived organizational support on job performance *via* workplace loneliness.

Variables	Effect	Standard error	95% confidence interval
Low extraversion	0.076	0.043	[−0.009, 0.160]
High extraversion	0.195	0.058	[0.082, 0.307]
Difference	0.118	0.043	[0.028, 0.201]

### Supplementary analyses

To further examine the mediating role of two dimensions of workplace loneliness in the relationship between perceived organizational support and job performance, the bootstrapping analyses employing M4 in PROCESS (Hayes, 2013) were conducted, generating 5,000 samples, and both emotional deprivation and social companionship were included as mediators. Results indicate that the indirect influence of perceived organizational support and job performance *via* emotional deprivation was not significant (indirect influence = 0.030, SE = 0.030, BC 95% CI = [−0.029, 0.093], included zero), while the indirect influence of perceived organizational support on job performance *via* social companionship was significant (indirect influence = 0.100, SE = 0.049, BC 95% CI = [0.004, 0.199]).

Considering social companionship, but not emotional deprivation, serves as a mediator in the association between perceived organizational support and job performance, we only examined the moderating role of extraversion in the relationship between social companionship and job performance and in the indirect influence of perceived organizational support on job performance *via* social companionship. As indicated in Model 9 of Table 5, the interaction between social companionship and extraversion had a significantly negative influence on job performance, which provided support for the moderating role of extraversion. Then, the bootstrapping analyses using Model 14 in PROCESS (Hayes, 2013), generating 5,000 samples, were conducted to examine the moderating role of extraversion in the mediating role of social companionship linking perceived organizational support to job performance, as shown in Table 6. It revealed a significant moderated mediation effect (the contrast = 0.127, SE = 0.048, BC 95% CI = [0.023, 0.211], did not include zero). Particularly, for individuals with low extraversion, the indirect influence of perceived organizational support on job performance *via* social companionship was not significant (indirect effect = 0.079, SE = 0.046, BC 95% CI = [−0.014, 0.167], included zero); while, for individuals with high extraversion, the mediating role of social companionship relating perceived organizational support to job performance was significant indirect effect = 0.206, SE = 0.065, BC 95% CI = [0.073, 0.326], which is in accordance with the data in Table 6; other than indirect effect = 0.206, SE = 0.048, BC 95% CI = [0.023, 0.211].

**Table 5:** Results of hierarchy regressions on the moderating role of extraversion in the relationship between social companionship and job performance.

Variables	Job performance			
	Model 1	Model 7	Model 8	Model 9
Gender	0.01	0.02	0.03	0.03
Age	−0.15^*^	−0.08	−0.07	−0.06
Tenure	0.41^**^	0.27^**^	0.25^**^	0.24^**^
Education	0.01	0.00	0.00	0.00
Position	0.04	0.00	0.00	−0.02
Social companionship		−0.40^**^	−0.37^**^	−0.41^**^
Extraversion			0.11^*^	0.16^**^
Social companionship *Extraversion				−0.19^**^
*R* ^2^	0.13	0.27	0.28	0.31
Δ*R*^2^	0.13	0.14	0.01	0.03
Δ*F*	9.64^**^	63.55^**^	4.20^*^	14.08^**^

**Table 6:** Results of moderating role of extraversion in the indirect influence of perceived organizational support on job performance *via* social companionship.

Variables	Effect	Standard error	95% confidence interval
Low extraversion	0.079	0.046	[−0.014, 0.167]
High extraversion	0.206	0.065	[0.073, 0.326]
Difference	0.127	0.048	[0.023, 0.211]

## Discussion

The present research examined the influence of perceived organizational support on workplace loneliness. Previous research has established the influence of interactions with others on one’s perception of loneliness in the workplace (Öge et al., 2018; Arslan et al., 2020; Kloutsiniotis et al., 2022). However, our study is among the first to examine the influence of perceived organizational support on loneliness, highlighting how connection with the organization contributes to the satisfaction of employees’ need for belonging. Moreover, we investigate the mediating role of workplace loneliness in the relationship between perceived organizational support and job performance. Supporting our arguments, perceived organizational support was negatively linked to workplace loneliness, thus leading to better job performance. The finding contributes to better understanding on the underlying mechanism through which perceived organizational support influences job performance.

The present research also indicates extraversion functions as an important personal trait that affects employees’ response to poor social ties in the workplace, such that the negative relationship between workplace loneliness and job performance was stronger for those higher on extraversion. Moreover, extraversion also moderates the mediating role of workplace loneliness linking perceived organizational support to job performance. Specifically, the indirect influence of perceived organizational support on job performance *via* workplace loneliness was only true of extravertive workers; meanwhile, this relationship was not significant for introvertive employees. Supplementary analyses also indicated that extraversion enhanced the negative relationship between social companionship and job performance, as well as the mediating role of social companionship relating perceived organizational support to job performance.

### Theoretical contributions

The findings of the present research make significant contributions to the literature on workplace loneliness and its antecedents and outcomes. First, our findings extend nascent literature on the antecedents of workplace loneliness, by examining how employees’ connection to the organization (referring to perceived organizational support) functions as significant ways to satisfy employees’ need for affiliation. Drawing from the COR theory, human resource co-exist in ecological systems that they would influence each other (Hobfoll, 2011). As shown in this paper, individuals’ interactions with the organization contribute to their need for close social contacts, which is in accordance with the work of Wayne et al. (2002), claiming that perceived organizational support is positively related to leader-member exchange. This finding provides additional support for the tenets of COR theory, and contributes to a new view for exploring the antecedents of workplace loneliness.

Second, the results indicated that workplace loneliness is an intervening mechanism linking perceived organizational support to job performance. To be more specific, social companionship functions as a mediator in the relationship between perceived organizational support and job performance, while the mediating role of emotional deprivation is not significant. Although Chiang and Hsieh (2012) have noted that perceived organizational support is positively linked to job performance, while, the work of Ozcelik and Barsade (2018) has indicated that workplace loneliness impedes job performance. In the present study, we confirmed that workplace serves as a mediator bridging perceived organizational support and job performance. In other words, perceived organizational support contributes to the satisfaction of individuals’ basic need for belonging, thus leading to better job performance. This is consistent with prior empirical studies that drew on the COR theory to investigate the mediating role of workplace loneliness relating transformational leadership to burnout (Kloutsiniotis et al., 2022). This conclusion supplements the evidence of a mediated mechanism relating perceived organizational support to performance and contributes to the expansion of COR theory application to the literature on workplace loneliness.

Thirdly, we examined the moderating role of extraversion in the relationship between workplace loneliness and job performance, and in the mediating role of workplace loneliness linking perceived organizational support and job performance. Although Mishra et al. (2018) found that the positive association between loneliness and depression was stronger for students with high extraversion than for those with low extraversion, scant attention has been paid to the moderating effect of extraversion in the influence of workplace loneliness on individuals in organizational psychology (Mishra et al., 2018; Ozcelik and Barsade, 2018). In response to this call, we focused on the moderating role of extraversion in the relationship between workplace loneliness and job performance. More importantly, extraversion also serves as a moderator in the indirect influence of perceived organizational support on job performance *via* meaningfulness. It seems to be because extraverted individuals had a higher need for belonging and more enjoy intimate interpersonal interactions, and they would be more anxious and more likely to employ avoidant emotional or behavioral coping strategies when suffering from insufficient social contact in the work context (Hogan, 1982; Depue and Collins, 1999). This finding is consistent with the emerging literature on the moderating role of personal traits in the influence of poor interpersonal relationships on individuals and enterprises (e.g., Mohapatra et al., 2020; Andel et al., 2021; Hu et al., 2021). Inferring from the SCT, not all individuals are equally influenced by the same emotional experiences (Bandura, 1991; Bandura, 2001). This finding provides additional evidence on SCT and extends its application to literature on workplace loneliness, indicating that personal traits function as moderators in the influence of emotional experiences (Bandura, 1991; Bandura, 2001). The present research is among the first to investigate the moderated mediation mechanism between perceived organizational support and job performance.

### Practical implications

The results of this study revealed that perceived organizational support is negatively related to one’s perceptions of loneliness in the workplace. In this vein, enterprises are recommended to enhance employees’ perceptions of being valued or cared for by their organization, such as by providing some rewards for employees’ organizational citizenship behaviors or other extra-role behaviors, and offering more training opportunities or implementation of flexible work schedules (Eisenberger et al., 1997; Chiang and Hsieh, 2012; Tian et al., 2021), to reduce their perceptions of loneliness in the workplace.

Enterprises will gain additional benefits from decreasing employees’ perceptions of workplace loneliness. Supervisors can organize social and networking activities to cultivate a harmonious, interactive, corporate culture to reduce employees’ feeling of loneliness, so as to enhance the positive association between perceived organizational support and job performance (Hu et al., 2021).

In addition, the results indicate that extraverts and introverts respond differently to workplace loneliness, such that the indirect influence of perceived organizational support on job performance *via* workplace loneliness is more prevalent for extraverted employees. Thus, enterprises and managers should be aware of the moderating effect of extraversion and take it into consideration in managerial practices (Mishra et al., 2018). Much attention should be paid to alleviating extraverts’ perceptions of workplace loneliness, given its stronger mediating effect in relating perceived organizational support to job performance.

### Limitations and future research

There are some limitations of the present study that need to be further examined in future research. First, although a two-wave survey was employed and the results of Harman’s one-factor test and CFA indicated that the common method bias was not significant in this study, it remains a limitation because of the same source of data. Future studies are recommended to invite leaders to evaluate employees’ job performance or employ archival data of performance, so as to minimize the influence of the common method variance.

Second, as subjective feelings, both perceived organizational support and workplace loneliness are dynamic processes. In this study, both of them are measured at a given time, which may have some influence on the reliability of the findings. Therefore, experimental or longitudinal designs can be employed to examine whether the fluctuation or duration of perceived organizational support and workplace loneliness makes a difference, which could further support the results of this study (Shadish et al., 2002; Holgado-Tello et al., 2016).

Third, there may be other variables that influence employees’ perceptions of loneliness in the workplace which were not included in the present research, such as approachableness (Ozcelik and Barsade, 2018), work at home, working hours, and work independently or interdependently (Zhang and Chen, 2022). Future studies are recommended to control these variables when investigating the mediating role of workplace loneliness relating personal or organizational resources to performance.

Finally, we only examined the moderating role of extraversion in the relationship between workplace loneliness and job performance and in the indirect influence of perceived organizational support on job performance *via* workplace loneliness. Consciousness may also function as a moderator in such relationship. As conscientious employees are more self-disciplined, obedient (Barrick and Mount, 1991), and better able to control their behaviors when suffering from negative emotions, thus they were less scratched by poor social ties in the workplace. In addition, self-construal may also make a difference in the relationship among perceived organizational support, workplace loneliness, and job performance, since individuals with interdependent self-construal tend to attach more importance to harmonious interpersonal relationship and are more affected by social rejection than those with independent self-construal (Markus and Kitayama, 1991). Future studies are recommended to examine whether conscientiousness, self-construal or other personal traits would serve as moderators in the relationship among perceived organizational support, workplace loneliness, and job performance.

## Conclusion

The present study contributes to better understandings of the moderated-mediation mechanism between perceived organizational support, workplace loneliness, extraversion, and job performance. It is indicated that workplace loneliness partially mediates the association between perceived organizational support and job performance, and extraversion serves as a moderator in such relationships. In particular, the indirect influence of perceived organizational support on job performance *via* workplace loneliness was stronger for individuals high in extraversion. Supplementary analyses revealed that two dimensions of workplace loneliness, social companionship, but not emotional deprivation, mediates the relationship between perceived organizational support and job performance; extraversion enhanced the relationship between social companionship and job performance, as well as the indirect effect of perceived organizational support on job performance *via* social companionship.

## Data availability statement

The raw data supporting the conclusions of this article will be made available by the authors, without undue reservation.

## Ethics statement

This study was approved by the Ethics Committee on Human Experimentation of Xi’, an University of Finance and Economics adhering to the Declaration of Helsinki. In the first-wave questionnaire, we introduced the research purposes and explained that this study welcomed voluntary participation and the data would only be used for research purposes. Informed consent was obtained from all participants included in the study before response to the questionnaire.

## Author contributions

GT designed the study, collected the data, and wrote the manuscript under the guidance of TL. TL contributed to data analysis and revision of the manuscript. RY contributed to the research design and revisions. All authors agreed on the journal to which the article will be submitted, gave final approval of the version to be published, and agreed to be accountable for all aspects of the work.

## Conflict of interest

The authors declare that the research was conducted in the absence of any commercial or financial relationships that could be construed as a potential conflict of interest.

## Publisher’s note

All claims expressed in this article are solely those of the authors and do not necessarily represent those of their affiliated organizations, or those of the publisher, the editors and the reviewers. Any product that may be evaluated in this article, or claim that may be made by its manufacturer, is not guaranteed or endorsed by the publisher.
